# Wolfram Syndrome 1: From Genetics to Therapy

**DOI:** 10.3390/ijerph19063225

**Published:** 2022-03-09

**Authors:** Luciana Rigoli, Valerio Caruso, Giuseppina Salzano, Fortunato Lombardo

**Affiliations:** 1 Department of Human Pathology of Adulthood and Childhood G. Barresi, University of Messina, 98125 Messina, Italy; giuseppina.salzano@unime.it (G.S.); fortunato.lombardo@unime.it (F.L.); 2 Psychiatry 2 Unit, Clinical and Experimental Medicine Department, University of Pisa, 56126 Pisa, Italy; valeriocaruso79@gmail.com

**Keywords:** Wolfram syndrome 1, *WFS1*, diabetes mellitus, optic atrophy, diabetes insipidus, sensorineural deafness, new therapies for Wolfram syndrome 1

## Abstract

Wolfram syndrome 1 (WS1) is a rare neurodegenerative disease transmitted in an autosomal recessive mode. It is characterized by diabetes insipidus (DI), diabetes mellitus (DM), optic atrophy (OA), and sensorineural hearing loss (D) (DIDMOAD). The clinical picture may be complicated by other symptoms, such as urinary tract, endocrinological, psychiatric, and neurological abnormalities. WS1 is caused by mutations in the *WFS1* gene located on chromosome 4p16 that encodes a transmembrane protein named wolframin. Many studies have shown that wolframin regulates some mechanisms of ER calcium homeostasis and therefore plays a role in cellular apoptosis. More than 200 mutations are responsible for WS1. However, abnormal phenotypes of WS with or without DM, inherited in an autosomal dominant mode and associated with one or more *WFS1* mutations, have been found. Furthermore, recessive Wolfram-like disease without DM has been described. The prognosis of WS1 is poor, and the death occurs prematurely. Although there are no therapies that can slow or stop WS1, a careful clinical monitoring can help patients during the rapid progression of the disease, thus improving their quality of life. In this review, we describe natural history and etiology of WS1 and suggest criteria for a most pertinent approach to the diagnosis and clinical follow up. We also describe the hallmarks of new therapies for WS1.

## 1. Introduction

Wolfram syndrome 1 (WS1; MIM 222300) is a rare autosomal recessive neurodegenerative disease first described in 1938 by Wolfram and Wagener [1]. The main clinical features are diabetes insipidus (DI), diabetes mellitus (DM), optic atrophy (OA), and deafness (D), hence the acronym DIDMOAD. However, WS1 is frequently complicated by other symptoms, such as urinary tract, endocrinological, psychiatric, and neurological abnormalities [2,3]. Early-onset non-autoimmune insulin-dependent DM and bilateral OA are key clinical criteria for the diagnosis of WS1 [1]. WS1 is a rare type of DM and has been included in subcategory 5A16.1 of the International Classification of Disease (ICD-11) [4]. Prognosis is poor, as the clinical course of WS1 is rapidly progressive and leads to a premature death of patients at the mean age of 30 years (25–49 years). The main cause of death is respiratory failure due to brainstem atrophy [5,6]. There are currently no therapies for WS1. However, careful clinical follow-up and supportive care can be helpful for relieving severe and progressive symptoms of WS1.

## 2. Epidemiology of WS1

WS1 is a very rare neurodegenerative disease. Epidemiological studies showed a prevalence of 1 in 770,000 [5] and 1 in 500,000 in children [7] in the UK; 1 in 100,000 in North America [8]; 1 out of 710,000 in the Japanese population [9]; and 0.74 out of 1,000,000 in the Italian population [10]. The highest prevalence of WS1 is of 1 in 68,000 in the Lebanese population [11] and of 1 in 54,478 in a population from a small area of Sicily (Italy) [12], probably due to the high rates of consanguinity in these populations [11,12]. The frequency of the WS1 carriers is not well known. However, a study in the UK population showed that WS1 carrier frequency was 1/354 [5].

WS1 is frequently not recognized early, as insulin-dependent non-autoimmune DM is the first clinical manifestation. It has been found that the prevalence of WS1 in patients with DM ranges from 0.57% in the UK [5] to 4.8% in the Lebanese population [11]. Zmyslowska et al. found that in pediatric insulin-dependent DM populations, WS1 was diagnosed with a delay of at least 7 years as WS1 patients were initially misdiagnosed as having type 1 DM [13]. Moreover, Lombardo et al. found that WS1 had a prevalence of 1 in 22.3 in Sicilian (Italy) patients with juvenile-onset, insulin-dependent DM aged <30 years [12].

## 3. Genetics of Wolfram Syndromes

**Figure 1:**
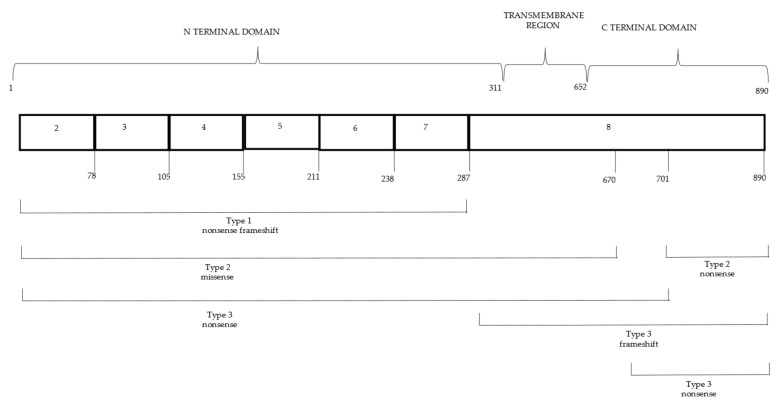
Genotypic classification of *WFSI* mutations.

The human WS1 gene (WFS1) was identified in 1998 [14,15]. It is located on chromosome 4p16, consists of eight exons, and encodes wolframin, a transmembrane glycoprotein of 890 amino acids (aa) in the endoplasmic reticulum (ER). Wolframin consists of nine transmembrane segments and a large hydrophilic region at both ends [16]. *WFS1* is highly expressed in brain tissue, pancreatic β-cells, heart, lung, and placenta [17]. Thus far, over 200 mutations have been found in the *WFS1* gene, and most of them are in exon 8 (https://www.ncbi.nlm.nih.gov/clinvar/, accessed on 10 February 2022) [18]. The region of exon 8 encodes the transmembrane and C-terminal domain of wolframin, which is important for the functionality of this protein [14,19]. *WFS1* mutations are frequently inactivating (nonsense or frameshift) [18], and most of them are transmitted in an autosomal recessive mode. However, autosomal dominant mutations have been found in WS-like diseases, such as *WFS1*-related non-syndromic low-frequency sensorineural hearing loss (LFSNHL) [20,21]. Specifically, *WFS1* mutations have been also implicated in non-syndromic hearing loss DFNA6/14/38 [22]. The great number of *WFS1* mutations, the complexity of the clinical picture of WS1, and the small number of patients (30–60 patients) do not allow a genotype-phenotype correlation [18]. De Heredia et al. analyzed both genetical and clinical data from 412 WS1 patients published since 1998 and found 178 mutations in *WFS1* [23]. The mutations were classified according to their effect on WFS1 expression, and it was suggested that patients with mutations causing absent wolframin production were more likely to have earlier onset diabetes mellitus, and perhaps earlier onset of optic atrophy, than patients with residual wolframin expression. Rigoli et al. studied 44 WS1 patients of Italian ethnicity and 1 Arab male (Morocco). In this study, mutations were subdivided into three groups according to predicted functional consequences, as the high genetic heterogeneity of WFS1 complicated genotype-phenotype correlations [10]. WS1 patients with nonsense mutations and frameshift and/or multiple amino acid insertion/deletions in both alleles resulting in absence of wolframin were included in group 1. Group 2 consisted of WS1 patients with missense mutations and/or single amino acid insertions in both alleles. Most of *WFS1* variants included in group 2 result in milder degradation of wolframin than those in group 1. Compound heterozygous WS1 patients with mutations not found in groups 1 and 2 were included in group 3. It was found that the age of onset of DM, D, and DI but not of OA differed between the three groups. Furthermore, the survival time of patients in group 1 tended to be shorter than that of patients in the other groups. The type of clinical manifestations of the WS1 patients was not different among the 3 groups. The results of the study in Italian patients suggest that there may be a genotype-phenotype correlation in WS1. The genetic and clinical study of a larger number of WS1 patients could elucidate the pathogenetic mechanisms of WS1 [10] (Table 1, Figure 1).

A second rare and neurodegenerative type of WS has been described, namely WS2, which is transmitted in an autosomal recessive mode. WS2 is caused by mutations in CDGSH iron-sulfur domain-containing protein 2 (*CISD2*) gene, which maps to chromosome 4q22-q23 and consists of three exons [24]. *CISD2* encodes the zinc-finger protein named “small intermembrane endoplasmic reticulum protein” (ERIS), which is highly expressed in tissues such as pancreas and brain [25]. Although the function of ERIS is not fully known, it has been found that it transfers iron to the mitochondria and thus is important for the regulation of iron and reactive oxygen species (ROS). Furthermore, ERIS plays a central role in the regulation of mitochondrial homeostasis and exchanges between ER and mitochondria and in the activation of autophagy and apoptosis [26]. The clinical features that characterize WS2 are still not completely established, as there are few affected subjects. The main symptoms of WS2 are ulcers of the upper intestine, mucocutaneous bleeding, and defective platelet aggregation, which are pathognomonic of WS2 and which are absent in WS1 [27,28]. They have been found in over 90% of patients affected by WS2. Therefore, they are useful clinical criteria for a differential diagnosis with WS1. Juvenile onset DM, variable degrees of OA, high-frequency sensorineural hearing impairment, DI, neurological and psychiatric abnormalities, endocrine disorders, and impaired renal function have also been reported. OA is progressive and is associated with loss of ganglion cells, but it is milder and less progressive than that of WS1. It has been suggested that optic nerve involvement is consistent with a diagnosis of optic neuropathy and not of optic atrophy [28].

## 4. Physiology and Pathophysiology of WS1

Wolframin is in the endoplasmic reticulum (ER) membrane, which plays a key role in the ability of cells to properly fold and post-translate secretory and the ER transmembrane proteins [29,30,31]. Mutations in *WFS1* cause an accumulation of misfolded proteins in the ER and therefore ER stress. High levels of misfolded proteins stimulate the unfolded protein response (UPR), which induces transcriptional and translational events that restore ER homeostasis. When ER stress is chronically persistent due to physiological processes (biosynthesis post-prandial of insulin) or to pathological processes (cancer, inflammatory diseases, viral infection, gene mutations), the UPR stimulates cell apoptosis. [17,29,31,32,33]. For this reason, high levels of ER stress found in WS1 cause apoptosis of pancreatic cells and alterations of neuronal cells [33].

UPR activates three transmembrane proteins located in the ER that function as sensors of stress: inositol-requiring protein 1 (IRE1), protein kinase RNA (PKR) -like ER kinase (PERK), and activating transcription factor 6 (ATF6). These transducers play a key role in survival adaptation and cell death processes. Moreover, some studies have found that, under physiological conditions, ER chaperones, such as immunoglobulin binding protein (BIP), maintain their luminal domains in a state of inactivity. BIP is released to facilitate the folding of accumulated proteins when high levels of UPR occur in ER [34,35,36].

Under conditions of physiological stress, IRE1 oligomerization and autophosphorylation occurs [37]. Subsequently, the RNase domain of IRE1 induces a splice of the X-binding protein 1 (XBP-1) mRNA because of which a transcriptionally active mRNA (sXBP-1) is formed. sXBP-1 is activated to XBP-1, a transcription factor. After translocation to the nucleus, XBP-1 upregulates some UPR target genes to re-establish protein homeostasis and activate cellular protection. Under pathological conditions, hyperactivation of IRE1 with consequent apoptosis is found. Moreover, IRE1 affects the biosynthesis of insulin: in hyperglycemia, it induces β cell homeostasis and thus an improvement in pro-insulin biosynthesis [38].

The transmembrane PERK protein is also involved in intricate mechanism of ER stress. Indeed, it activates the phosphorylation of eIF2alfa, a eukaryotic initiation translation factor 2alfa. The role of eIF2alfa is to decrease ER biosynthetic activity and to enhance the translation of both ATF4 transcription factor and apoptosis-antagonizing transcription factor (AATF) mRNAs. ATF4 activates genes involved in amino acid transport and metabolism, glutathione biosynthesis, and antioxidant responses. Moreover, ATF4- ATF3-CHOP axis promotes apoptosis. Thus, under conditions of pathological ER stress, apoptosis is induced by the continuous activation of these factors. On the other hand, AATF promotes the survival of cells [39].

ATF6 is the third important regulator of the UPR response. BIP dissociation, induced by ER stress, promotes translocation of ATF6 to the Golgi apparatus. Here, the cleavage of ATF6 occurs by means of some proteases with subsequent formation of a cytosolic active transcription factor. Upon activation, ATF6 translocates to the nucleus where it improves protein folding, processing, and degradation activity by upregulating ER transcriptional homeostatic factors. A regulatory role of ATF6 in lipid biosynthesis has also been described [40,41,42].

The intricate pathway of UPR is negatively regulated by *WFS1*. Under physiological ER stress, *WFS1* negatively regulates ATF6, reduces the activation of the ER stress response element of the ER (ERSE) promoted by ATF6, and induces the stabilization of E3 ubiquitin ligase HRD1 (HMG-CoA reductase degradation protein) and thus the suppression of stress signals. [17]. Conversely, in WS1, the hyperactivation of ATF6 promotes both the expression of genes involved in apoptosis, such as CHOP, ATF4, BIP, and sXBP1, and the reduced gene expression of insulin. Moreover, wolframin regulates the calcium release and absorption mechanisms in the ER. It is a calmodulin (CaM) with several functions, including the interaction with many cellular proteins and the regulation of the Ca2 + signal transduction processes involved in apoptosis [43]. High levels of ER stress have been shown to cause alterations in mitochondrial function, thus suggesting that WS1 could be a mitochondrial disease [44]. Recently, a link between ER stress, increased cytosolic Ca2+levels, impairment of mitochondrial dynamics, and inhibition of neuronal development in WFS1-deficient neurons has been described [45,46]. In healthy cells, WFS1 is linked to neuronal calcium sensor 1 (NCS1) and inositol 1,4,5-trisphosphate receptor (IP3R) to induce transfer of Ca2+ between ER and mitochondria. In *WFS1*-deficient cells, a severe decrease of NCS1 levels was found, which causes a reduction of ER-mitochondria interactions and transfer of Ca2+ [47]. Therefore, there is a strong causal link between ER stress, alterations in cytosolic levels of Ca 2+ and in mitochondrial dynamics, and developmental delay in *WFS1*-deficient neuronal cells [45]. In this intricate pathogenic mechanism, the alterations of mitochondria-associated ER membranes (MAMs) play an important role [48,49]. MAMs are dynamic domains of interaction between mitochondria and ER in which several proteins involved in UPR are located. The role of these proteins is to stabilize the structure of MAMs and to facilitate the functional dialogue between ER and mitochondria. Indeed, MAMs facilitate the transfer of Ca2 + between ER and mitochondria mainly through IP3R [46,49]. According to these observations, Cagalinec et al. suggested that a “mitochondrial phenotype” in WS1 patients could be due to severe alterations of mitochondrial dynamics caused by even mild ER stress [46]. Zmyslowska et al. studied a human WS cell model in which skin fibroblasts reprogrammed into induced pluripotent stem cells (iPS) and then into neural stem cells (NSCs) were subjected to induced ER stress. The analysis of the proteins involved in mitochondrial function showed a down-regulation of the subunits of the respiratory chain complexes, an upregulation of the proteins involved in the Krebs cycle, and mechanisms of glycolysis in WS NSC cells. These alterations were not found in the control cells. These data have shown that severe mitochondrial damage resulting in functional and morphological alterations of mitochondria plays a key role in the pathogenesis of WS1 [50].

Finally, *WFS1* was found to regulate the function of the sarco/endoplasmic reticulum Ca^2+^-ATPase (SERCA), an important protein implicated in β-cell ER calcium homeostasis [51,52].

## 5. Natural History and Clinical Manifestations

The clinical diagnosis of WS1 requires the coexistence of two main criteria: early onset of insulin-dependent non-autoimmune DM (DM) (usually during the first decade of life) and bilateral optic atrophy (OA) before age 15 [2]. Diabetes insipidus (DI) and sensorineural hearing loss (D) are usually associated with DM and OA. Thus, WS1 has also been defined with the acronym DIDMOAD. Other clinical manifestations of WS1 are renal tract abnormalities or neuropsychiatric disorders [23,53]. Many studies have shown that renal anomalies are very frequent in WS1. Thus, the acronym DIDMOADUD has been suggested [2,10]. Other symptoms include cognitive problems and mood disorders [5,54]. As WS1 is characterized by many clinical features, it has been suggested that WS1 can be diagnosed in the following cases: (1) coexistence of the two major criteria (DM + OA); (2) one main criterion together with two minor criteria; and (3) two of any of the DIDMOAD manifestations [2,10,23]. Many studies have attempted to establish the order in which symptoms of WS1 start [10,55]. However, it is hard to establish an accurate order of the WS1 clinical manifestations, as this syndrome is very rare, and therefore, the number of patients that may be studied is small. De Heredia et al. analyzed the clinical and genetic features of 412 published WS1 patients with age specified for any clinical symptom. They found that DM (98.21%) and OA (82.14%) were the most frequent clinical features. D and DI were shown in 48.21% and 37.76% of cases, respectively. Other clinical manifestations, such as renal anomalies (19.39%) and neurological symptoms (17.09%), were found in a smaller number of WS1 patients. The mean age of death was about 30 (range 25–49) years. Interestingly, the mean age of death showed two peaks of higher frequency, one at 24 years and the other at 45 years. Respiratory failure was the most frequent cause of death [23] (Table 2).

## 6. Insulin-Dependent and Non-Autoimmune Diabetes Mellitus

DM is typically the first clinical feature of WS1, with onset at the mean of 6 years (3 weeks–16 years). Insulin-dependent DM of WS1 differs from common type 1 DM (T1D) in the following features: earlier diagnosis, rarely positive autoantibodies, rare ketoacidosis, longer remission periods, lower daily requirement of insulin, mean values of HbA1c lower than T1D, and frequent episodes of hypoglycemia [10,23,56]. Moreover, slowly progressing microvascular complications, such as microvascular retinopathy, are less common than T1D. It has been suggested that the impaired carbohydrate metabolism is a consequence of neurological alterations caused by ER stress. [56,57,58]. Therefore, clinicians must consider these clinical differences between WS1 DM and T1D to avoid a misdiagnosis of WS1 because such a mistake would have very severe health repercussions on the patient.

## 7. Optic Atrophy

In WS1 patients, OA is diagnosed in the first decade of life, usually before 15 years of age. The first clinical manifestation of OA is the progressive decrease of visual acuity with loss of color vision. The blindness develops after a few years [23,58,59]. Ophthalmological anomalies, such as cataract (29.6–66.6%), alterations in pupillary reflexes to light, nystagmus, maculopathy, and glaucoma, have been found in rare cases [59]. Pigmentary retinopathy is very rare, and few cases have been reported [6,60,61,62,63]. Pigmentary maculopathy in WS1 patients, although rare, may be due to the severe alterations of mitochondrial dynamics that have been described in WS1 [64]. Microspherophakia was found in two sisters who were also affected by congenital cataract, glaucoma, and OA [65]. Many ophthalmic alterations, which also include abnormalities of the retinal nerve fiber layer thickness, were found in 15 WS1 patients at relatively early stages [62]. However, Zmyslowska et al. showed that alterations of retinal nerve fiber layer thickness are less frequent in WS1 subjects than in T1D patients or healthy subjects [57]. Waszczykowska et al. found a significant reduction of corneal sensitivity in patients with WS1. Indeed, the corneal nerve fiber, branch density, and nerve fiber length were low in WS1, suggesting corneal nerve degeneration. In addition, the variability of corneal sensitivity was found to correlate with the degree of disease progression [66].

Full eye examination by assessing of visual acuity and color vision, fundoscopy, visual field, and optical coherence tomography (OCT) scan should be done early. The visual evoked potential test allows to evaluate the therapeutic efficacy. Other expedients are increase in the size of the image and writing on mobile devices, such as computers, notebooks, smartphones, and tablets, and the use of voice systems. Unfortunately, there are no drugs available to treat OA. Attempts have been made to slow the progression of OA using drugs, such as idebenone or docosahexaenoic acid, but there are few data about the efficacy of this therapy [67,68]. 

## 8. Diabetes Insipidus

Central DI is frequent, affecting approximatively 70% of WS1 patients [5]. It occurs at a mean age of 14 years (3 months–40 years). However, a high variability in age of onset was found, as DI is often diagnosed with delay. Assessment of DI should be done by urine concentration test, which is recommended for all patients with DM; color vision impairment; deafness; and neurological symptoms. In most cases, intranasal or oral administration of desmopressin improves the clinical picture of DI [18].

## 9. Sensorineural Deafness

Sensorineural deafness (D) occurs at a mean age of 12.5 years (range 5–39 years) in 62% of WS1 patients [5]. The clinical spectrum is broad as the severity of hearing impairments varies between patients. The progression of D is relatively slow and first affects the high frequencies [5]. In WS1 patients, D is more severe than in other patients with hearing loss due to degenerative impairments in the central nervous system [5]. Annual or two-year audiometric testing and brain stem auditory response (ABR) assessment are useful for monitoring D in WS1 patients. Therapeutic tools, such as hearing aids and cochlear implants, are very helpful for WS1 patients [69]. Hearing symptoms in WS1 must be carefully evaluated as low-frequency sensorineural D caused by dominant mutations of WFS1 has been described [70]. However, patients suffering from this dominant type of genetic deafness have a different clinical picture than WS1.

## 10. Neurological and Psychiatric Manifestations

Most WS1 patients (>60%) have been shown to exhibit neurological symptoms at a mean age of 40 years (range 5–44 years) [5], but in some cases, onset is earlier [10,71]. De Heredia et al. found the onset of neurological complications at the mean age of 23 years, with two peaks of greater frequency, one at 13 years and the other at 30 years [23]. Cerebellar ataxia of the trunk is the most common manifestation (45%), and a neurological counseling one or two times a year is recommended [55]. Other neurological abnormalities are peripheral neuropathy (39%), cognitive impairment (32%), epilepsy (26%), and lastly, dysarthria, dysphagia, and nystagmus (10%) [55]. Severe complications, such as aspiration pneumonia, can be prevented by swallowing therapy. Esophageal dilatation and esophagomyotomy are useful in some cases. Neurological symptoms, such as loss of the gag reflex, decreased ability to taste and smell, orthostatic hypotension, anhidrosis, hypohidrosis or hyperhidrosis, constipation, gastroparesis, hypothermia, or hyperpyrexia, may often be reported [55]. Atrophy of the brain, cerebellum, and brainstem are abnormalities found by nuclear magnetic resonance imaging (MRI) in 54% of WS1 patients [55]. Respiratory failure or dysphagia are common causes of mortality [5,55]. Thus far, the progression of neurological manifestations cannot be slowed down, as there is no therapy. Psychiatric disorders, such as severe depression with suicide attempts, psychosis, sleep abnormalities, verbal impulsivity, and physical aggression, can complicate the clinical picture in WS1 patients. Moreover, a predisposition to psychiatric diseases was found in *WFS1* heterozygotes [72]. Cognitive performance is generally normal. However, Chaussenot et al. found that 32% of 59 studied WS1 patients had cognitive impairment [55]. It has been suggested that the smell and sleep alterations can be used as indicators to follow-up WS1 patients with psychiatric manifestations [73].

## 11. Urological Abnormalities

Neurogenic bladder, which causes hydroureteronephrosis, urinary incontinence, and recurrent infections, has frequently been found in WS1 patients [55]. Urinary tract abnormalities have been found in up to 90% of patients. The average age of onset is 20 years old, and specifically, three high-frequency peaks were found: one at 13 years, the second at 21, and the third at 33 years [23].

Neurogenic bladder and upper urinary tract dilation are the main urological abnormalities. Anticholinergic drugs and clean intermittent catheterization are therapeutic tools for neurogenic bladder [23]. Some WS1 patients also undergo electrical stimulation and physiotherapy [2]. Follow-up is carried out through clinical, instrumental, and laboratory checks of renal function. Moreover, the measurement of the residual urinary volume after voiding by ultrasound and urodynamic tests are needed. Urinary tract infections at a very early age are the first manifestations of WS1 only in rare cases [74]. Urine culture is required in WS1 patients suffering from fever or other symptoms, such as headache. Yuca et al. described a Turkish family in which the course of chronic renal failure was rapidly progressive in some WS1-affected members [75].

## 12. Endocrinology and Reproductive Biology

Primary and secondary hypogonadism, more frequent in males, are the main manifestations of endocrine function impairment in WS1 patients. Delayed menarche and menstrual cycle alterations are frequent in WS1 females, but ovarian function is normal, and some pregnant patients have been described [59]. Short stature, growth hormone (GH) deficiency, and impaired corticotrophin secretion were found in WS1 patients [5] due to anterior pituitary hypofunction of hypothalamic origin [59]. Growth rate and pubertal development must be closely monitored for GH therapy, and steroid supplementation should be considered during stressful periods, such as severe infections [76].

## 13. Additional Anomalies

Gastrointestinal disorders include gastroparesis (29%), bowel dysmotility (24%), and bowel incontinence [71]. Congenital heart diseases, such as Fallot’s tetralogy and pulmonary valve stenosis, have been reported in rare cases of WS1 [59,77,78], and hence, heart monitoring is recommended.

## 14. Diagnosis of WS1

A careful and accurate diagnosis of WS1 allows for early identification of patients so that appropriate interventions can be initiated. History and clinical manifestations, such as the diagnosis of OA after that of DM under the age of 16, should lead to suspicion of WS1. Visual abnormalities and insulin-dependent DM must be carefully evaluated in WS1 patients, as they may be misdiagnosed as T1D with diabetic retinopathy. This mistake would cause a delay in diagnosis of WS1 [2,13]. Given the clinical complexity of WS1, alterations such as DI, sensorineural D, and neurological and urological symptoms together with non-auto-immune insulin-dependent DM or OA, allow to suspect WS1. Differential diagnosis should be made with mitochondrial diseases, deafness caused by *WFS1* mutations, autosomal dominant OA, Bardet–Biedl syndrome, Alström syndrome, and Friedreich ataxia [59]. Genetic tests, such as exome sequencing and genome sequencing-based diagnostic methods, are valuable tools to confirm or rule out the diagnosis of WS1. Sequencing of the entire *WFS1* with all eight exons and their flanking intronic regions is recommended [10,79]. Early diagnosis of WS1 is imperative to enable successful follow-up that includes specialist consultations and appropriate instrumental and laboratory tests. If WS1 is suspected, thorough genetic counseling should be done to study family members even if they are asymptomatic.

## 15. New Chances for Therapies

ER stress plays a key role in the pathogenesis of WS1 as shown in many clinical and genetic studies [17,39,45]. Unfortunately, there are currently no therapies available to delay, block, or reverse WS1 progression. Drugs for WS1 therapy should target ER stress alterations and therefore should act by modulating ER stress, regulating ER calcium homeostasis, and cellular proteostasis [80,81].

## 16. Chemical Chaperones

Shang et al. developed an experimental model in which pluripotent stem cells (iPSCs) obtained from WS1 patient skin cells were used to generate β cells. iPSCs are very useful tools for studying potential new drugs for WS1, as they are characterized by high levels of ER stress and reduced insulin content. Recently, chemical chaperones have been suggested for WS1 therapy. They are a class of molecules that play an important role in ER protein folding. Indeed, they stabilize native conformation during folding of *WFS1* mutant proteins [82]. Therefore, chemical chaperones improve the function of β cells and prevent cell death through a reduction in ER stress. Food and Drug Administration (FDA) has approved two chemical chaperones, namely 4-phenylbutyric acid (PBA) and tauroursodeoxycholic acid (TUDCA) [81]. It has been shown that PBA regulates insulin synthesis. Chemical chaperones use the same ER stress-reducing mechanism also to slow neurodegeneration in patients with WS1 [80]. Recent studies have shown that AMX0035, a molecule being investigated in ASL, reduces neuronal death and dysfunction by targeting ER stress. FDA have designed AMX0035 as an *orphan drug*. Preclinical data have suggested that AMX0035 is a promising drug to stop irreversible changes in OA in WS1 patients. These studies suggest that chemical chaperones can delay the progression of WS1 and preserve the remaining tissue functions [81,82]. Recently, preclinical model studies have shown that activation of the sigma-1 receptor (S1R) chaperone, an ER-resident protein involved in calcium ion transfer, restored the function of MAMs due to wolframin deficiency. In particular, the agonist S1R PRE-084 restored calcium transfer, improved mitochondrial respiration, and modified autophagy and cellular mitophagy, thus ameliorating symptoms in zebrafish and rodents. These data suggest that S1R chaperone may be a potential drug for the therapy of WS1 [83]. Although they are a promising class of therapeutic drugs for WS1, the need to use high concentrations of chemical chaperones limits their use. Therefore, high-throughput screening (HTS) has been proposed to identify small molecules of chaperones that protect neuronal and pancreatic β-cells against ER stress [84].

## 17. ER Calcium Stabilizers

It has been found that the therapy with calpain inhibitor XI and ibudilast preserve cytosolic calcium as well as cell viability and glucose-stimulated insulin secretion of *WFS1*-KO cells [85]. In WS1, the depletion of ER calcium activates the calpain protease. Calpains are calcium-dependent cysteine proteases, typically regulated by changes in cytosolic calcium [86]. The activity of calpains is reversibly inhibited by calpain inhibitor XI, which normalizes resting cytosolic calcium in *WFS1*-KO cells [85]. Therefore, the calpain pathway could be a target for WS1 new drugs [68]. Ibudilast, an unselective phosphodiesterase inhibitor, blocks the cleavage of cAMP [80]. cAMP interacts with calcium signaling pathways and is implicated in insulin secretion of pancreatic β-cells [87,88]. It has been found that ibudilast normalizes calcium levels through its interaction with NCSI [85,89]. This drug has already been approved for use in some human diseases [90], and it may be a safe candidate for therapy of WS1. FDA approved drug dantrolene sodium has also been proposed for WS1 therapy as it has been shown to stabilize ER calcium levels (Clinical Trials.gov identifier: NCT028029268). Until now, it has been used for the treatment of malignant hyperthermia and muscle spasms. Dantrolene suppresses calcium efflux from the ER to the cytosol, as it targets the ryanodine receptor located in the ER membrane. Thus, cell death and neurodegeneration are prevented [68].

Other molecules were suggested to maintain ER homeostasis. ER calcium homeostasis is regulated by SERCA ATPase [51,52], and therefore, drugs that activate SERCA ATPase and maintain high ER calcium levels have been suggested for WS1 therapy. Focus has been put on IP3R, which could be used as a regulator calcium channel drug [52] because it regulates the release of calcium from the ER. It has been found that pioglitazone inhibits IP3R, and thus, it could be used as an ER calcium stabilizer. In *WFS1* knock-out mice, Akiyama et al. showed that pioglitazone protects from β-cell death and prevents the development of DM [91]. Indeed, it is a drug for the therapy of type-2 DM (T2D). However, pioglitazone causes severe adverse effects, and this slows its use for therapy of WS1 [92]. Rapamycin, an immunosuppressive drug, could also be used to reduce calcium levels. However, like pioglitazone, it has negative side effects [93].

Toots et al. proposed carbachol, a muscarinic receptor 3 (M3) agonist, as a drug for the therapy of DM in WS1 patients, as it potentiates glucose-stimulated insulin secretion. Acetylcholine binds to M3 muscarinic receptors, thus producing IP3 and diacylglycerol. By binding of IP3 with IP3Rs on the ER membrane, there is mobilization of intracellular calcium, an increase of the levels of cytoplasmic Ca^2+^, and thus an enhancement of glucose-stimulated insulin secretion [94]. Recent studies have identified a new calcium stabilizer called JVT-519, which plays a key role in ER, as it binds to the ryanodine receptor 2 (RyR2), stabilizing it in its closed state. Urano et al. are evaluating the efficacy of JTV-519 in cell and mouse models of WS1. Moreover, they are conducting a clinical trial in WS1 patients (https://grantome.com/grant/NIH/UH2-TR002065-01 accessed on 20 February 2022).

## 18. Targeting ER Stress

The p21 (cip) protein, a cyclin-dependent kinase inhibitor with anti-apoptotic effect, has been suggested for future WS1 therapy. It plays a role in cell proliferation and survival after ER stress. Gharanei et al. found that the p21 (cip) level was reduced in *WFS1* mutant cells and that there was an inverse association between p21Cip1 expression and cell death. Valproic acid (VPA), a mood stabilizer, seems to increase the expression of p21Cip1, thus protecting WS1 cells against cell death [95]. Some studies have shown that VPA is neuroprotective, as it inhibits the apoptosis induced by ER stress, has neurotrophic effects, and promotes neurite outgrowth [96,97]. The efficacy of valproate in delaying neurodegeneration and visual alterations is recently being evaluated (ClinicalTrials.gov identifier: NCT03717909).

Glucagon-like peptide-1 receptor (GLP-1R) agonists have been suggested as drugs for WS1 therapy due to their antidiabetic effect, which has been shown in both animal models and WS2 patients [44,98,99]. After meals, intestinal L cells secrete a peptide called GLP, which reduces ER stress-mediated pancreatic cell death and promotes growth and survival of β cell [98]. It was found that GLP-1R agonists prevent and suppress ER stress-mediated apoptosis in WS1 [64]. Moreover, recent studies on two WS1 rodent models have shown that GLP-1R agonists improve DM in WS1 [98,99,100]. Kondo et al. showed that 24-week therapy with liraglutide, a long-acting GLP-1R agonist, improved glycemic control and reduced daily insulin dose by 20% in one patient with WS1 [98]. It has been hypothesized that therapy with liraglutide decreases ER stress, inflammation, and proliferation in pancreatic β-cells, preventing or delaying DM [100]. Moreover, as GLP-1R is widely found in neuronal cells, it has been suggested that it has a protective role in both central and peripheral nervous systems [101,102]. Seppa et al. found that 6-month liraglutide therapy leads to a reduction of neuroinflammation and to an improvement of ER stress in the inferior olive in WS1 rats. Finally, liraglutide appears to protect retinal ganglion cells from apoptosis and the optic nerve from degeneration [103]. However, WS1 therapy with GLP-1R agonists is not yet well known [104,105,106]. It has been hypothesized that dipeptidyl peptidase-4 (DPP-4) inhibitors such as gemigliptin, sitagliptin, or vildagliptin could be used for WS1 therapy. Indeed, DPP-4 deactivates GLP 1-R, resulting in increased GLP-1 levels and improved DM [107].

## 19. Mitochondrial Modulators

Recently, it has been hypothesized that molecules acting on alterations in mitochondrial dynamics, such as mitochondrial modulators, can be used as drugs for the therapy of WS1 [46,47]. Although this hypothesis is interesting, so far, there are few studies on the therapeutic efficacy of mitochondrial modulators.

## 20. Other Therapeutic Approaches

There are currently numerous attempts for WS1 therapy involving the use of FDA-approved drugs or new therapies that target specific pathophysiological stages in the development of WS1. Recently, many studies have suggested gene and regenerative therapies for the replacement of damaged tissues, such as pancreatic β-cells, and neuronal and retinal cells [108].

## 21. Gene Therapy

Urano et al. have used skin cells from WS1 patients to induce pluripotent stem cells (iPSCs) by adeno-associated viral systems (AAVs). The goal was to correct mutations in *WFS1* gene and to differentiate the iPSCs into neurons, retinal cells, and pancreatic β-cells for transplantation [108].

Recently, Clustered Regularly Interspaced Short Palindromic Repeats (CRISPR) technology has been used to replace pathogenic *WFS1* mutations with wild-type *WFS1* alleles in WS1 patient iPSCs and thereby to create iPSC-derived organoids. This therapeutic strategy could be associated with regenerative medicine [108].

## 22. Regenerative Medicine

In WS1, there is a need to replace damaged tissues, such as pancreatic β-cells, retinal ganglion, and neuronal cells. Therefore, a regenerative therapy with iPSCs has been developed [108]. Lu et al. generated iPSCs from WS1 patients and from their siblings and/or parents. It was hypothesized that iPSCs could be differentiated into progenitor of neural cells, cells of retinal ganglion, and pancreatic cells and be used for therapy tests and cell replacement therapy [68].

Moreover, it has been suggested that mesencephalic astrocyte-derived neurotrophic factor (MANF) could be used in regenerative medicine on the tissues mainly involved in WS1.

MANF, a regeneration factor produced by astrocytes, is a protein located in the ER, and its expression and secretion are stimulated by ER stress. It is a primary survival factor that activates the proliferation of pancreatic β-cells and protects against ER stress-mediated apoptosis [109,110,111]. Cytoprotective activity of MANF is unclear, as its receptor is unknown. MANF has been found to have a cell-surface receptor, namely neuroplastin (NPTN), and the MANF-NPTN complex seems to modulate cell death. Therefore, survival signal of MANF-NPTN could be a target for the therapy of diseases caused by ER stress [111]. Through AAV systems, MANF could be introduced into neurons, pancreatic β-cells, and retinal cells to suppress neurodegeneration and improve glucose tolerance and visual acuity [2]

The therapeutic strategies proposed for WS1, and their stages of development are illustrated in Table 3 and Table 4.

## 23. Conclusions

WS1 is a rare genetic syndrome with severe and widespread neurodegenerative clinical features. The clinical picture of WS1 require a multidisciplinary healthcare approach to be successfully treated. Prompt diagnosis allows identification of the early symptoms of WS1, thus favoring an improvement in morbidity and a reduction in mortality. Furthermore, genetic counseling is always needed in affected families. Many cell lines and animal model studies have highlighted the role of ER stress in WS1. Therefore, a thorough understanding of the pathogenetic mechanisms of WS1 could help to understand common diseases characterized by apoptosis caused by ER-stress, such as diabetes mellitus, neurodegenerative diseases, inflammatory diseases, and tumors. The understanding of the pathogenesis of these diseases may be useful to develop novel therapeutic strategies. Likewise, an extensive understanding of the relationships between ER stress, cytosolic Ca^2 +^ alterations, mitochondrial dynamics, and neurodevelopment in WS1 may also be useful for developing new drugs for WS1 therapy.

## Figures and Tables

**Table 1:** Functional alterations of wolframin according to type of *WFS1* mutations.

Class		Alterations of Wolframin Function
A absence of wolframin production	A1	Wolframin depletion due to degradation of *WFS1* mRNA
	A2	Wolframin depletion due to mRNA and protein degradation
	A3	Wolframin depletion due to degradation of protein
B		Reduced expression of a deficient wolframin
C		Expression of a deficient wolframin

**Table 2:** Clinical features of patients with Wolfram syndrome 1.

Major Clinical Features	Age at Diagnosis	Percentage of WS1 Patients with a Single Disorder
Diabetes insipidus	14 years (3 months–40 years)	37.7%
Diabetes mellitus	6 years (3 weeks–16 years)	98.2%
Optic atrophy	11 years (6 weeks–19 years)	82.1%
Sensorineural hearing loss	12.5 years (5–39 years)	48.2%
Neurological and autonomic disorders (central apnea, ataxia, dysphagia, areflexia, epilepsy, decreased ability to taste and detect odors, headaches, orthostatic hypotension, hyperpyrexia, hypothermia, constipation, gastroparesis)	16 years (5–44 years)	17.1%
Urinary tract complications (neurogenic bladder, bladder incontinence, urinary tract infections)	20 years (13–33 years)	19.4%
**Other common clinical features**		
Psychiatric symptoms (anxiety, panic attacks, depression, mood swings, sleep abnormalities, psychosis)	20.5 years (17–23 years)	44.4%
Endocrinological disorders (hypogonadism, deficient growth hormone secretion, corticotropin deficiency, delayed menarche in female)	8 years (7–9 years)	6.6%

**Table 3:** Therapeutic strategies proposed for Wolfram syndrome 1.

**Chemical chaperones** (4-phenylbutyric acid and tauroursodeoxycholic acid)	Improvement of β-cell functions by action on ER stress cell. Stabilization of native conformation during folding of mutant *WFS1* proteins [81]. Slowdown of neurodegeneration in WS1 [80]
**ER calcium stabilizers**	
Calpain inhibitor XI	Normalization resting cytosolic calcium [85]
Ibudilast	Block of the cleavage of cAMP, which interacts with the calcium pathway [80,81]. Normalization of the levels of calcium by interaction with NCS1 [85,89].
Dantrolene	Blockade of the ryanodine receptor in the ER membrane and subsequent suppression of calcium efflux from ER to the cytosol [68]
Drugs that activate SERCA ATPase	Maintenance of high levels of ER calcium [51,52]
Pioglitazione	Inhibition of IPR3-mediated release of calcium from the ER (91)
Rapamycin	Reduction of the cytoplasmic calcium levels [93]
Carbachol	Mobilization of calcium intracellular, enhancement of glucose- stimulated insulin secretion [94]
**Drugs targeting ER stress**	
Valproic acid	Protection against apoptosis, induction of *WFS1* expression, modulation of the ER stress response [96,97]
**GLP-1R agonists**	
Liraglutide, exenatide, semaglutide	Prevention and suppression of ER stress-mediated cell death. [98,99,100,101,102]
Inhibitors of dipeptyl peptidase- 4 (DPP-4): Gemigliptin, sitagliptin, vildagliptin	Increase of GLP-1 levels [107]
**Mitochondrial modulators**	Restoration of mitochondrial functions [108]
**Gene Therapy**	
AAV rescue by WT *WFS1*	Transfection of wild-type *WFS1* into retinal ganglion cells of WS1 patients to produce a correct protein [101]
CRISPR/Cas9 mutant WFS1 gene editing	Replacement of mutant *WFS1* with-wild type *WFS1* alleles in WS1 patients’ iPSCs [108]
**Regenerative Medicine**	
Cell-replacement therapy (iPSCs)	Replacement of WS1 damaged tissues, such as pancreatic β-cells, retinal Ganglion, and neuronal cells, by iPSCs [68,108]
Regenerative gene delivery (MANF)	Activation of the proliferation of pancreatic β-cells, protection against ER stress mediated apoptosis, and suppression of neurodegeneration [2,109,110,111].

**Table 4:** Development stages of therapeutic strategies proposed for Wolfram syndrome 1.

Chemical Chaperones	Phases of the Therapeutic Approaches
AMX0035	Clinical phase
HTS (high-throughput screening)	Under study
**ER calcium stabilizers**	
NCS1/Ibudilast	Preclinical studies
Dantrolene	Phase Ib/IIa non-randomized, open-label, clinical trial (Clinical Trials.gov identifier: NCT028029268)
2end Generation Calcium Stabilizer JTV-519	Clinical phase
**Drugs targeting ER stress**	
Valproic acid	Phase III randomized, double-blind, placebo-controlled trial (Clinical Trials.gov identifier: NCT03717909).
GLP-1R agonists/liraglutide	Clinical trial off-label phase I
**Gene Therapy**	
AAV rescue by WT *WFS1*	Preclinical studies
CRISPR/Cas9 mutant WFS1 gene editing	Preclinical studies
**Regenerative Medicine**	
Cell-replacement therapy (iPSCs)	Preclinical studies
Regenerative gene delivery (MANF)	Preclinical studies
